# Transcriptome analysis of scions grafted to potato rootstock for improving late blight resistance

**DOI:** 10.1186/s12870-021-03039-w

**Published:** 2021-06-15

**Authors:** Yuexin Li, Degang Zhao

**Affiliations:** 1 College of Life Sciences, Guizhou University/Agricultural Bioengineering Institute, Guiyang, 550025 China; 2 Key Laboratory of Plant Resources Conservation and Germplasm Innovation in Mountainous Region (Ministry of Education), Guizhou University, Guiyang, 550025 China; 3 Guizhou Plant Conservation Technology Center, Guizhou Academy of Agricultural Sciences, Guiyang, 550006 China

**Keywords:** Potato, Graft, Late blight, Transcriptome sequencing

## Abstract

**Background:**

Late blight seriously threatens potato cultivation worldwide. The severe and widespread damage caused by the fungal pathogen can lead to drastic decreases in potato yield. Although grafting technology has been widely used to improve crop resistance, the effects of grafting on potato late blight resistance as well as the associated molecular mechanisms remain unclear. Therefore, we performed RNA transcriptome sequencing analysis and the late blight resistance testing of the scion when the potato late blight-resistant variety Qingshu 9 and the susceptible variety Favorita were used as the rootstock and scion, respectively, and vice versa*.* The objective of this study was to evaluate the influence of the rootstock on scion disease resistance and to clarify the related molecular mechanisms.

**Results:**

A Kyoto Encyclopedia of Genes and Genomes pathway enrichment analysis revealed that the expression levels of genes related to plant–pathogen interactions, plant mitogen-activated protein kinase (MAPK) signaling pathways, and plant hormone signal transduction pathways were significantly up-regulated in the scion when Qingshu 9 was used as the rootstock. Some of these genes encoded calcium-dependent protein kinases (CDPKs), chitin elicitor receptor kinases (CERKs), LRR receptor serine/threonine protein kinases (LRR-LRKs), NPR family proteins in the salicylic acid synthesis pathway, and MAPKs which were potato late blight response proteins. When Favorita was used as the rootstock, only a few genes of late blight response genes were upregulated in the scion of Qingshu 9. Grafted plants using resistant variety as rootstocks inoculated with *P. infestans* spores showed significant reductions in lesion size while no significant difference in lesion size was observed when susceptible variety was used as the rootstock. We also showed that this induction of disease resistance in scions, especially scions derived from susceptible potato varieties was mediated by the up-regulation of expression of genes involved in plant disease resistance in scions.

**Conclusions:**

Our results showed that potato grafting using late blight resistant varieties as rootstocks could render or enhance resistance to late blight in scions derived from susceptible varieties via up-regulating the expression of disease resistant genes in scions. The results provide the basis for exploring the molecular mechanism underlying the effects of rootstocks on scion disease resistance.

## Background

Potato (Solanum tuberosum L.), an annual solanaceous herb native to the Andes in South America has become the fourth most important food crop in the world [1]. China has become one of the primary potato-producing countries since its introduction in the seventeenth century. Globally, China is the primary potato-producing country. Potato tubers are a rich source of nutrients, including starch, proteins, minerals, crude fiber, and anti-oxidative and anti-aging compounds. In addition to serving as a commonly consumed vegetable, potato is widely used in the textile, pharmaceutical, food, dye, paper, and other industries because of its high starch content. Thus, potato has extremely diverse uses, and its production can substantially affect national economies. However, potato yields are severely affected by pests and adverse environmental conditions. The most harmful threat to sustainable potato production worldwide is late blight caused by *Phytophthora infestans* (Mont.) De Bary [2]. Late blight has drastically decreased potato yields, including losses of up to 100% in some cases. Annual direct economic losses due to late blight infections worldwide are as high as US $6.7 billion, which corresponds to 15% of the total potato output [3]. Consequently, developing viable methods for preventing and controlling late blight during potato cultivation is critical. Favorita, which is a high-yielding potato variety that produces high-quality tubers, was introduced to China from the Netherlands in 1981 by the China-owned Assets Supervision and Administration Bureau of the Central Ministry of Agriculture. Favorita is a high-yielding potato variety that produces high-quality tubers and was introduced to China from the Netherlands. However, because it is susceptible to late blight, strict planting conditions are required during its cultivation. An outbreak of late blight will seriously impact the yield and quality of Favorita potatoes [4]. Therefore, there is a critical need for enhancing the late blight resistance of Favorita.

Grafting, which is an ancient agricultural technique dating back to 424 BC, is a vegetative hybrid cultivation method in which two cut plants are joined and allowed to heal to develop into a new plant. Buds or branches are usually used as the scion, whereas the root stem serves as the rootstock; the scion is attached to an incision in the rootstock, after which the fused plant materials heal to form a grafted plant [5]. In agriculture, grafting technology is mainly used to increase crop yield, improve the branching structure, and enhance crop resistance to biotic and abiotic stresses [6]. China has a long history of applying crop grafting technology. Specifically, it has been widely used to breed stress-resistant tomato, eggplant, pepper, and melon varieties as well as to elucidate the mechanisms underlying the stress resistance of these crops [7]. In studies using drought-tolerant tobacco varieties as rootstocks, Huo (2016) and other researchers concluded that grafted tobacco plants can improve their drought resistance by regulating antioxidant enzyme activities and stress-responsive gene expression [8]. Wang et al. (2015) grafted tomato to purple potato rootstock and observed that grafting significantly increased the tomato yield and decreased the incidence of bacterial wilt, without affecting fruit quality [9]. Additionally, grafting cucumber to Yunnan black-seed pumpkin rootstock can increase cucumber resistance to blight [10]. Therefore, choosing an appropriate rootstock can increase plant stress resistance and yield. Grafting is also relevant for studying long-distance signaling in plants [11]. Numerous studies proved that RNA, proteins, hormones, and even chloroplast and nuclear genomes can be transported from the rootstock to the scion [12–14]. Changes to plant traits may be closely related to the exchange of material between the scion and rootstock. However, the molecular basis of plant trait modifications and the physiological or biochemical changes after grafting remain unknown. Transcriptome analyses of grafted plants can reveal the specific genes involved in regulating the physiological responses induced by grafting [15] as well as the differentially expressed genes (DEGs) in the transcriptional network and main metabolic pathways influencing plant growth, development, and responses to environmental stresses before and after grafting to explore the mechanism and mechanism of the trait changes of grafted plants from the molecular level [16]. For example, it was improved that the plant hormones salicylic acid (SA), jasmonic acid (JA), and ethylene (ET) were important signaling molecules involved in abiotic and biotic stress responses. Genes related to disease responses and calcium-dependent signaling were also crucial for plant stress responses [17].

In this study, we used the early-maturing and susceptible potato variety Favorita (abbreviated as “F”) and the mid-late maturing and highly late blight-resistant potato variety Qingshu 9 (abbreviated as “Q”) as test materials. These varieties were grafted onto each other as scions and rootstocks. We conducted resistance tests on the scion after grafting. Moreover, on the basis of the potato genome sequence, we analyzed the transcriptome data for the scion to explore the effect of potato grafting on scion gene expression and late blight resistance.

## Results

### Resistance of potato to late blight after grafting

**Fig. 1:**
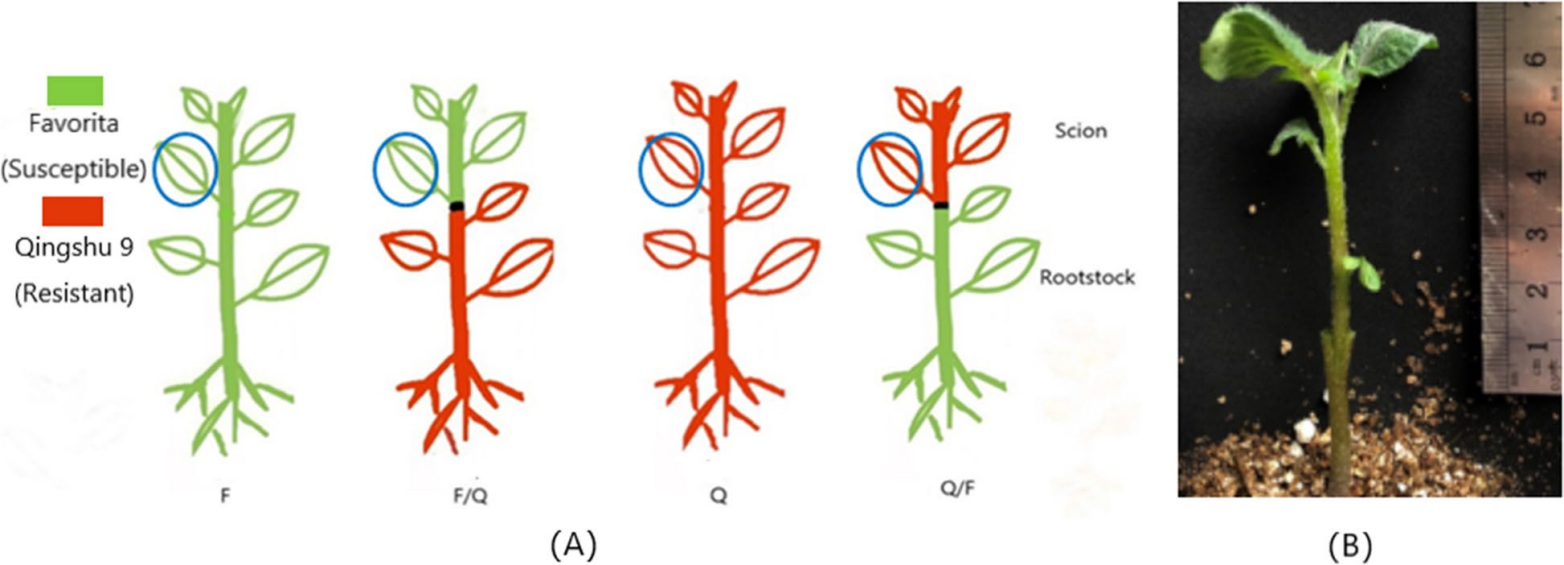
Late blight index results of the separated leaves after inoculating *P. infestans* on the 7th day. **a** The percentages of F, F/F and F/Q lesions in leaf area were 47.46 ± 5.69%, 38.29 ± 1.49%, 23.24 ± 2.03%, and the disease grades were grade 4, grade 4, grade 3 respectively. **b** The percentages of Q, Q/Q and Q/F lesions in leaf area were 4.85 ± 0.81%, 3.62 ± 0.75%, 5.32 ± 5.08%, and the disease grades were all grade 2

At 35 days after grafting, the F/F, F/Q, Q/Q, and Q/F scion leaves were collected for an in vitro inoculation assay, with leaves from ungrafted F and Q plants serving as controls. After grafting, the late blight resistance of susceptible variety F was significantly improved. When using the highly resistant variety Q as rootstocks, the proportion of diseased spots in susceptible variety was reduced by 51.03% and 39.31% respectively compared with the ungrafting and self-grafting, which were significant (*P* < 0.05). At the same time, the proportion of diseased spots of susceptible variety self-grafted was 19.3% lower than that of ungrafted, the difference was significant (*P* < 0.05). The percentages of F, F/F and F/Q lesions in leaf area were 47.46 ± 5.69%, 38.29 ± 1.49%, 23.24 ± 2.03%, and the disease grades were grade 4, grade 4, grade 3 respectively. Compared with Q, the proportion of Q diseased spots on scion of Q/F increased by 9.70%, the difference was not significant. Compared with Q/Q, the proportion of ungrafted Q disease spots decreased by 25.36%, the difference was not significant. The percentages of Q, Q/Q and F/Q lesions in leaf area were 4.85 ± 0.81%, 3.62 ± 0.75%, 5.32 ± 5.08% respectively, and the disease grade was 2 (Fig. 1).

### Transcriptome sequencing and assembly

**Fig. 2:**
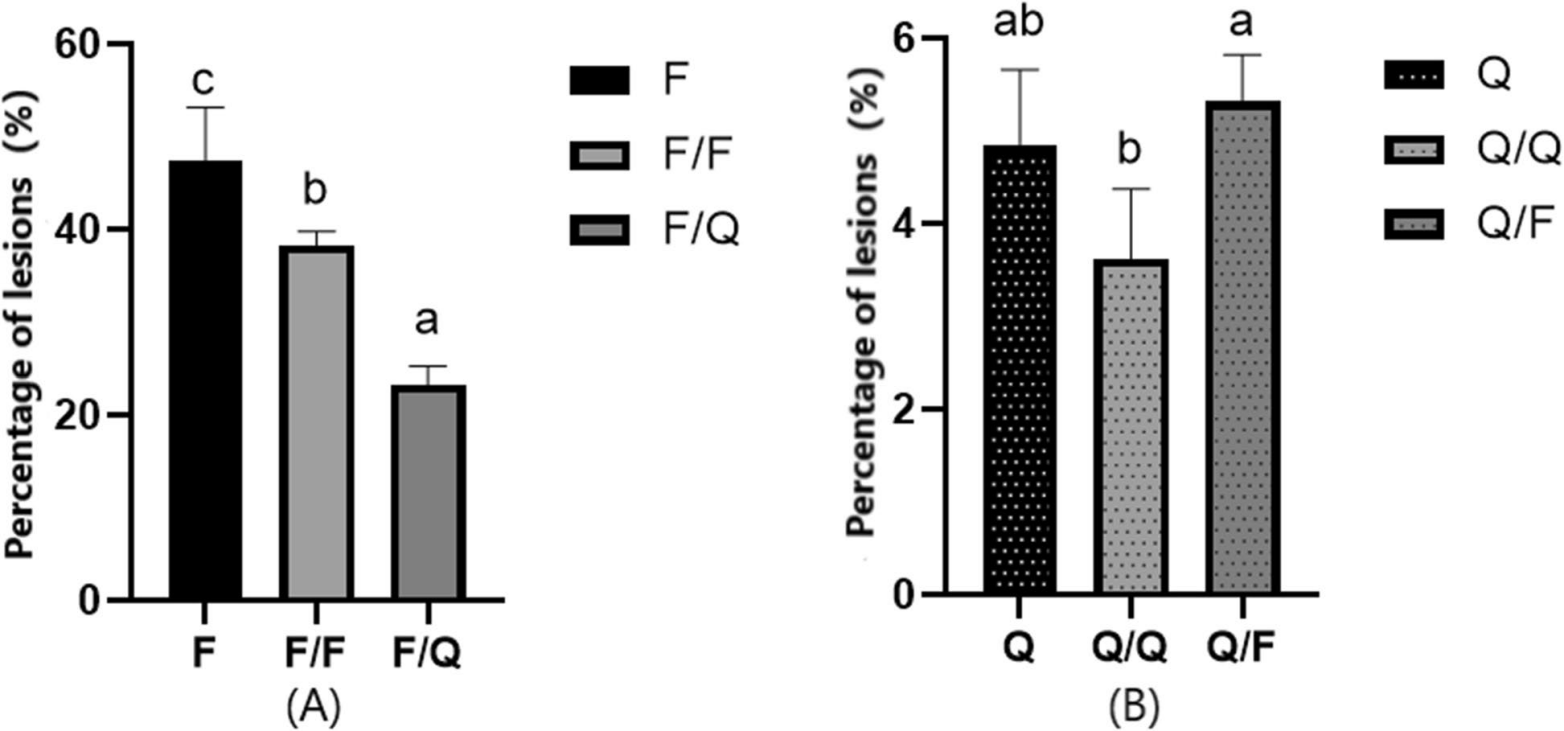
Diagram of F and Q grafting. **a** Illustration of F and Q grafting. The green plant is F and the red is Q. The position of the leaves collected for transcriptome sequencing is circled. **b** Image of a grafted plant. Healthy young shoots (4–5 cm) with 4–5 leaves were used as scions, healthy young shoots were cut 2–3 cm above the soil level to produce the rootstocks. A 0.7-cm deep vertical incision was made in the middle of the rootstock. The scion was cut into wedges, inserted into the incision. (Both images were drawn and photographed by the author.)

The transcriptomes of the third leaves from the top of the analyzed plants were sequenced with the Illumina high-throughput sequencing platform, there were 3 biological replicates for each sample were sequenced (Fig. 2a), resulting in 6.5 × 10^7^, 5.7 × 10^7^, 6.1 × 10^7^, and 5.5 × 10^7^ raw reads, which were filtered to obtain 6.3 × 10^7^, 5.6 × 10^7^, 6.1 × 10^7^, and 5.4 × 10^7^ clean reads for the F/Q, Q/F, F, and Q leaves, respectively. Overall data sequencing error rate was 0.03% for all leaves. Additionally, for the F/Q, Q/F, F, and Q leaves, the Q30 (i.e., the percentage of total bases with phred value greater than 30, where phred = -10log10 (e)) was 93.66%, 93.76%, 93.86%, and 92.82% and the GC content was 42.38%, 42.31%, 42.24%, and 42.18%, respectively. The clean reads were aligned to the reference genome sequence using the HISAT program (i.e., hierarchical indexing for spliced alignment of transcripts) [18]. The average mapping rate for the F/Q, Q/F, F, and Q leaves was 87.61%, 86.09%, 82.43%, and 85.78%, respectively. The similarity of the mapping rate among the samples indicated that the clean read data were comparable between samples. Therefore, the transcriptome sequencing results were reliable and appropriate for further analysis (Table 1).

**Table 1:** Summary of sequence reads for four RNA samples including two control groups (F, Q) and two grafted groups (F/Q, Q/F)

	Raw reads	Clean reads	Error_rate /%	Q30 /%	GC_pct /%	Total map /%	Unique map /%
F	6.1 × 10^7^	6.1 × 10^7^	0.03	93.86	42.24	82.43	79.68
Q	5.5 × 10^7^	5.4 × 10^7^	0.03	92.82	42.18	85.78	83.17
F/Q	6.5 × 10^7^	6.3 × 10^7^	0.03	93.66	42.38	87.61	85.25
Q/F	5.7 × 10^7^	5.6 × 10^7^	0.03	93.76	42.31	86.09	83.42

### Screening of DEGs

**Fig. 3:**
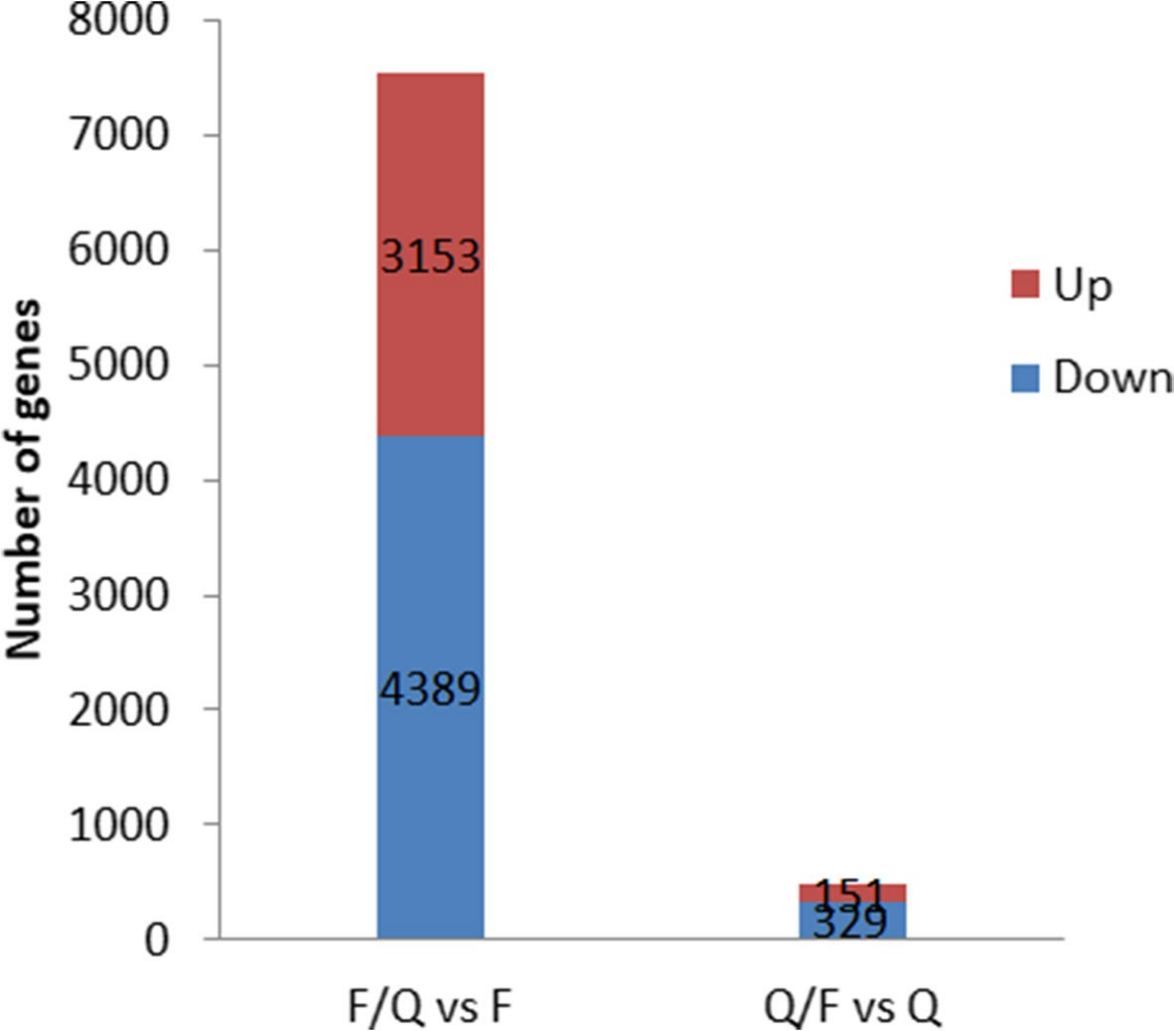
Number of DEGs detected between ungrafted and grafted potato seedlings (F *vs* F/Q and Q *vs* Q/F)

Using a gene expression level fold-change > 1 and a *p*-value < 0.05 as the criteria, we detected 8,022 DEGs (Fig. 3). The F/Q *vs* F comparison revealed 3,153 up-regulated genes and 4,389 down-regulated genes. In contrast, the Q/F *vs* Q comparison identified 329 up-regulated genes and 151 down-regulated genes.

### Gene Ontology (GO) enrichment analysis of DEGs

**Fig. 4:**
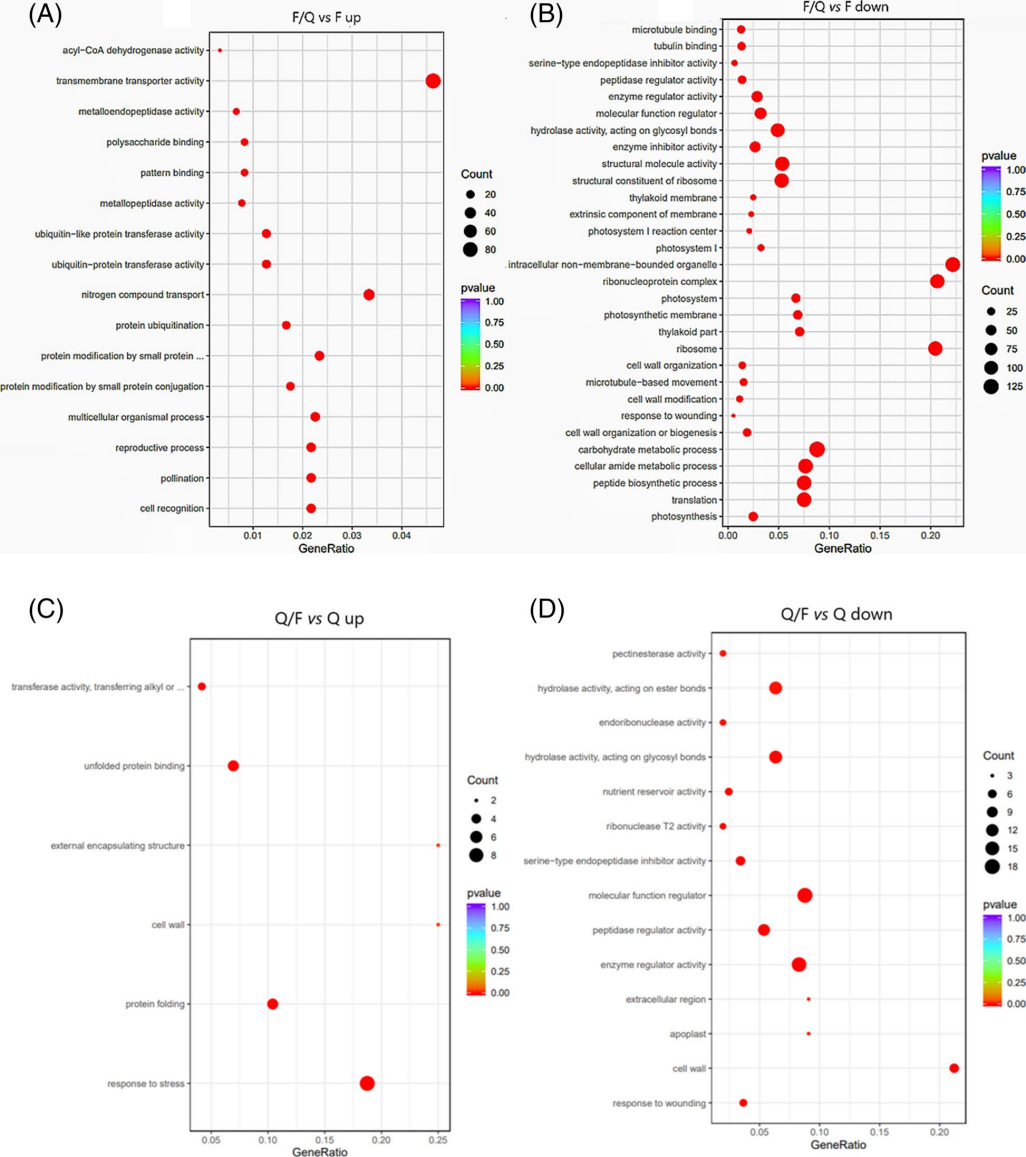
GO statistics and significant enrichment analysis of identified DEGs detected between ungrafted and grafted potato seedlings (*P* < 0.05). **a** and **b** Scatter plot of DEGs between F and F/Q. **c** and **d** Scatter plot of DEGs between Q and Q/F

The DEGs were functionally annotated based on GO classifications (Fig. 4), which divided the genes into the following three main categories: biological process (BP), cellular component (CC), and molecular function (MF). Compared with F, up-regulated genes in F/Q were significantly enriched in BP and MF. The most enriched in BP was nitrogen compound transport genes, and the most enriched in MF was transmembrane transporter activity genes. And the down-regulated genes were enriched in carbohydrate metabolic process, peptide metabolic process and photosynthesis in BP. In CC, they were mainly enriched in intracellular non-membrane organelles genes. In MF, structural molecule activity was the most enriched, and genes such as cell regulation and peptidase catalytic activity were also significantly down-regulated. Compared with Q, the up-regulated genes of Q/F were mainly enriched in the response to stress in BP, the cell wall and external encapsulating structure in CC, and the unfolded protein binding in MF. Most of the down-regulated genes were genes of cell regulation and peptidase catalytic activity in MF, and were also enriched in response to wouding in BP and cell wall in CC.

### Kyoto Encyclopedia of Genes and Genomes (KEGG) pathway enrichment analysis of DEGs

**Fig. 5:**
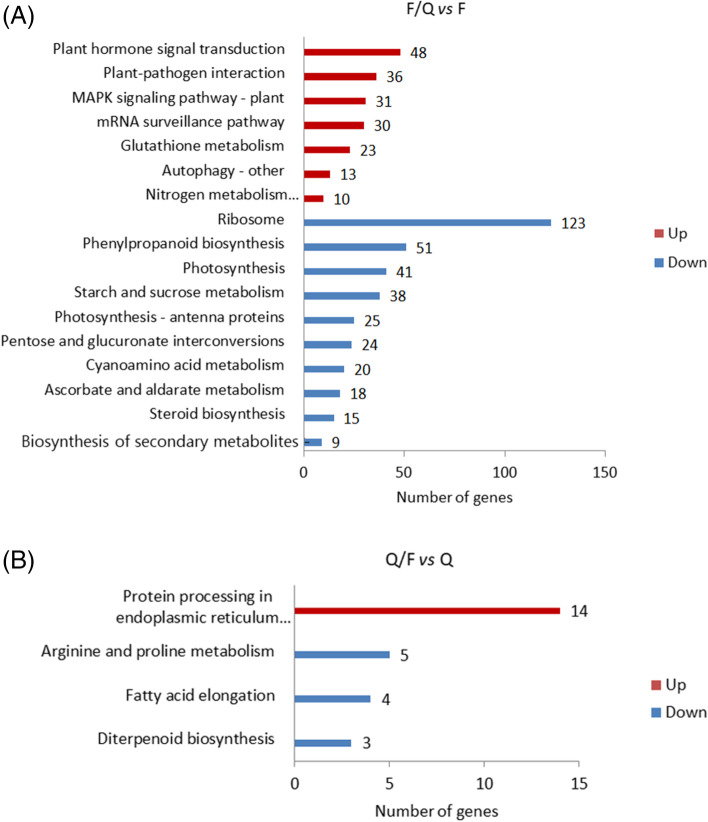
Significantly enriched KEGG pathways among DEGs detected between ungrafted and grafted potato seedlings (*P* < 0.05). **a** The DEGs between F and F/Q. **b** The DEGs between Q and Q/F

The DEGs identified in the two comparisons were also analyzed using the KEGG pathway database (Fig. 5). The top 20 enriched KEGG pathways were identified. More specifically, in the F/Q *vs* F comparison, the up-regulated DEGs were mainly associated with the mRNA monitoring pathway, autophagy—other, glutathione metabolism, plant–pathogen interactions, plant hormone signal transduction, nitrogen metabolism, and the mitogen-activated protein kinase (MAPK) signaling pathway, whereas the down-regulated DEGs were primarily associated with ribosomes and the photosynthesis antenna protein as well as with photosynthesis, phenylpropane biosynthesis, cyanoamino acid metabolism, steroid biosynthesis, and pentose and glucuronide conversions. Regarding the Q/F *vs* Q comparison, the up-regulated DEGs were mainly related to the protein processing pathway in the endoplasmic reticulum, whereas the down-regulated DEGs were involved in fatty acid elongation, arginine and proline metabolism, and diterpenoid biosynthetic pathways.

#### Effects of grafting on the expression of genes related to plant–pathogen interactions

**Fig. 6:**
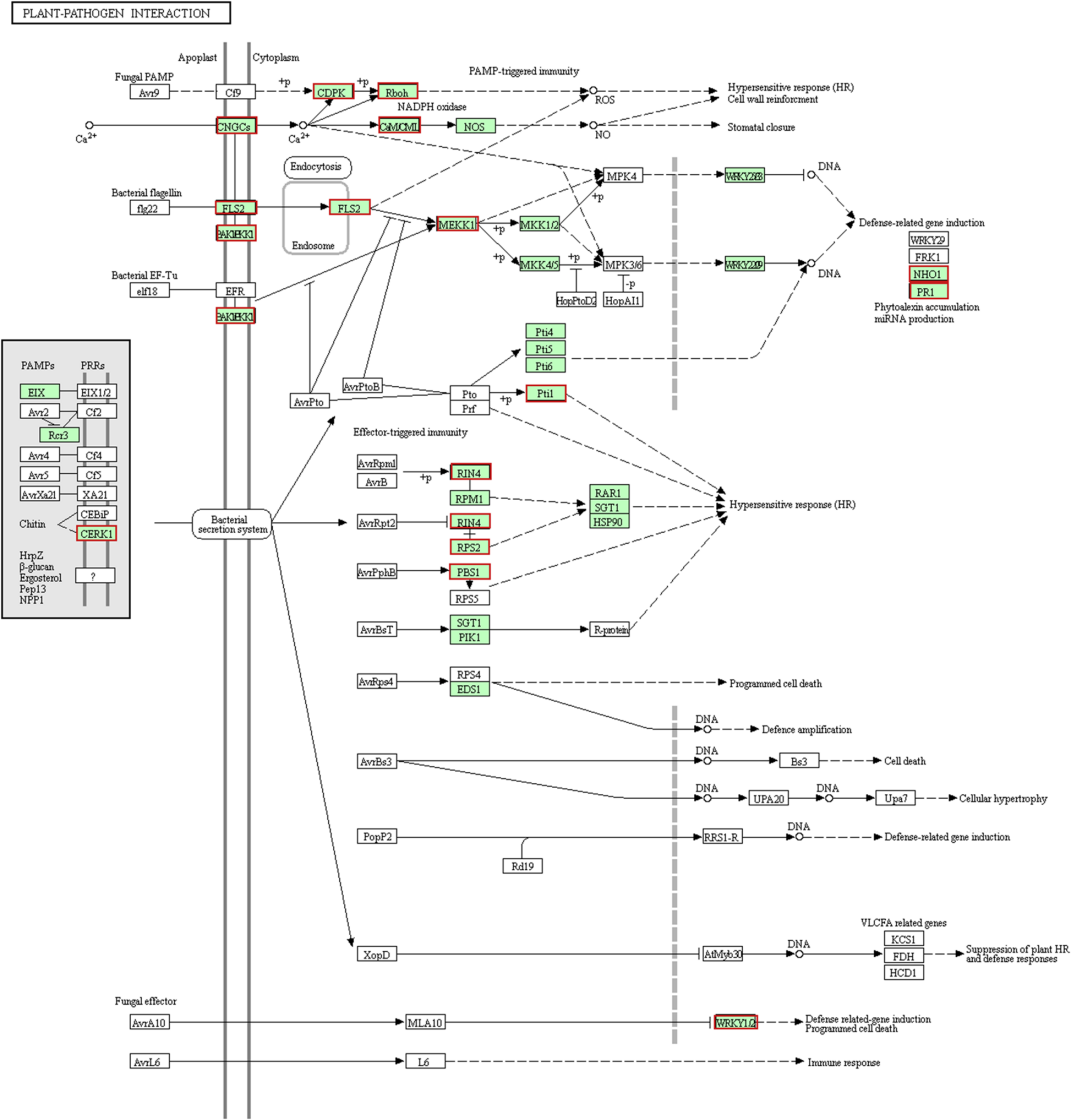
Up-regulated genes in the plant–pathogen interaction signaling pathway (F/Q *vs* F). The up-regulated genes are indicated by red boxes

The KEGG pathway analysis revealed that 36 plant–pathogen interaction-related genes were more highly expressed in the F/Q leaves than in the F leaves (Fig. 6). These genes encoded 10 calcium-dependent protein kinases (CDPKs), five cyclic nucleotide-gated ion channel (CNGC) proteins, three chitin elicitor receptor kinases (CERKs), two LRR receptor serine/threonine protein kinases (LRR-LRKs), and two WRKY transcription factors. On the basis of the results of the KEGG pathway analysis of the DEGs in the Q/F *vs* Q comparison, the expression levels of only three heat shock protein (HtpG) genes and a glutathione peroxidase (GSH-Px) gene were up-regulated.

#### Effects of grafting on the expression of genes involved in MAPK signaling pathways

**Fig. 7:**
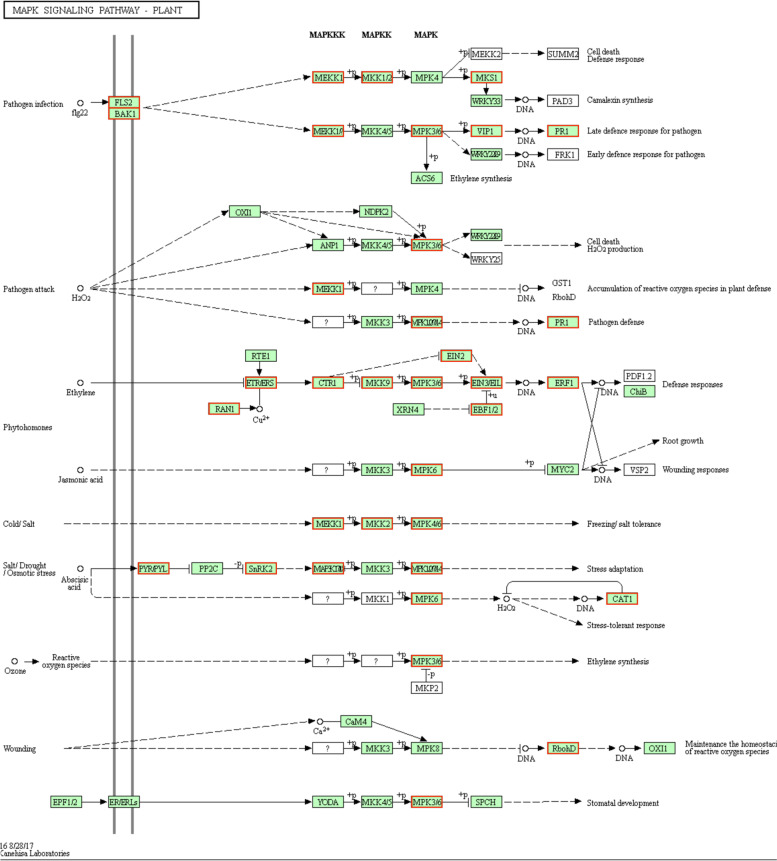
Up-regulated genes in the MAPK signaling pathway (F/Q *vs* F). The up-regulated genes are indicated by red boxes

The KEGG pathway analysis indicated that 31 MAPK signaling pathway-related genes had higher expression levels in the F/Q leaves than in the F leaves (Fig. 7). These genes included six encoding MAPKs, five encoding ethylene-insensitive proteins, and three encoding the abscisic acid (ABA) receptor PYL. Additionally, the DEGs more highly expressed in Q/F leaves than in Q leaves included a serine/threonine protein kinase (SRK2) gene and a protein phosphatase (PP2C) gene.

#### Effects of grafting on the expression of plant hormone signaling-related genes

**Fig. 8:**
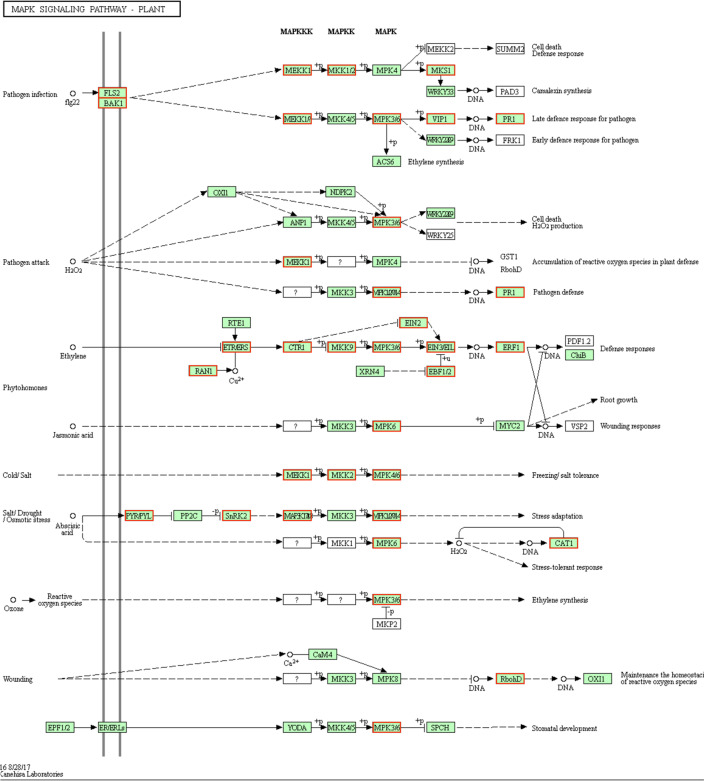
Up-regulated genes in the plant hormone signal transduction pathway (F/Q *vs* F). The up-regulated genes are indicated by red boxes

Plant hormone signal transduction is affected by grafting. In the F/Q *vs* F comparison, the up-regulated genes were mainly associated with the ET, SA, and JA pathways. Among the genes in the ET signal transduction pathway, the expression levels of one ETR gene, one EIN2 gene, four EIN3 genes, and one ERF1/2 gene were up-regulated. Six DEGs were related to the JA signal transduction pathway, of which four were up-regulated genes and two were down-regulated genes. The up-regulated genes encoded JA synthase, whereas the down-regulated genes were TIFY family genes. There were 11 up-regulated genes and one down-regulated gene involved in the SA pathway, including three NPR family genes, five TGA family genes, and two genes encoding disease-related proteins (Fig. 8). The up-regulated DEGs in the Q/F *vs* Q comparison encoded a SRK2 gene and a PP2C gene.

## Discussion

### Effects of grafting on potato late blight resistance

In order to explore the influence of the rootstock on late blight resistance of scion after grafting two different potato varieties, we used the potato late blight resistant variety Qingshu 9 and the susceptible variety Favorita for grafting. Then we tested the late blight resistance of the separated leaves of the scion. Our analysis indicated that both self-grafting of the late blight-susceptible variety F and grafting with highly resistant variety Q could improve the late blight resistance of the scion F. It showed that grafting itself could induce to improve the plant disease resistance. This may be related to the increased stress response, the activity of antioxidant enzymes and the expression of related genes after grafting. In addition, after grafting with disease-resistant rootstocks, there was an interaction between the rootstock and scion. The disease-resistant substances or genes in the rootstock may be passed upwards to the scion, thereby making it more resistant to disease. This is consistent with the conclusion of previous studies that the resistance of grafted plants may come from the upward conduction of disease-resistant substances in the rootstock on the one hand, and the resistance is induced and stimulated by grafting itself on the other hand [19, 20]. Besides, the late blight resistance of scion Q grafting with F was lower than that of self-grafting of Q. So we speculate that there are some materials exchange between the scion and rootstock which have the important influence on the resistance of scion late blight.

### Analysis of the effect of grafting on potato late blight resistance based on transcriptome sequencing data

To further explore the molecular mechanism of scion late blight resistance in grafted plants, the transcriptomes of the stable F/Q and Q/F scions were sequenced with the Illumina HiSeq 4000 sequencing platform, with ungrafted F and Q used as the controls. A total of 7,542 DEGs were identified in the F/Q *vs* F comparison, including 3,152 up-regulated genes and 4,389 down-regulated genes. The 480 DEGs revealed by the Q/F *vs* Q comparison consisted of 329 up-regulated genes and 151 down-regulated genes. The DEGs were functionally characterized based on the enriched GO terms. Most of the DEGs were related to wound responses, cell parts, responses to stimulation, biological regulation, and catalytic activities. The transcription of many genes related to cell rearrangements, cell division, the metabolic mode, and stress responses changed in the scion after grafting. The up-regulated genes in F/Q were enriched in transmembrane transporter activity, nitrogen compound transport and ubiquitin-protein transferase activity genes, and the up-regulated genes in Q/F were enriched in response to stress and transferase activity, transferring alkyl or aryl (other than methyl) groups genes. It showed that cell reorganization or maybe there were some substances transferred from rootstock to scion, leading to the expression of related genes was increased. In addition, after grafting, the cell wall was rebuilt, so expression of polysaccharide binding, cell wall, protein folding, and cell cortex part genes was up-regulated significantly.

The enriched KEGG pathways indicated that compared with the corresponding expression in the ungrafted F, the expression levels of some stress-related genes were significantly up-regulated in the F scion. These genes were mainly related to plant–pathogen interactions, plant MAPK signaling, and plant hormone signaling. This was consistent with the results of earlier KEGG pathway enrichment analyses of the DEGs identified after the transcriptome sequencing of grafted tomato scions by Wang Hui et al. and the DEGs revealed by the transcriptome sequencing of grafted litchi sections by Chen Zhen et al. [21, 22]. They believed that grafting induce the oxidative stress of plants, leading to the stimulation of the antioxidant defense system in the scion as well as the up-regulated expression of related genes. Besides the expression levels of genes related to auxin, gibberellin, ABA, ET, SA, and JA also changed. For example, the expression of (auxin inlux carrier) Aux1 family, (small auxin-up RNA) SAUR family, and transcription factor family genes which involved in the regulation of plant growth and development were up-regulated. We speculate that it was caused by cell reconstruction after grafting. At the same time, in the current study, we found that some of up-regulated genes encoded CDPKs, CERKs, LRR-LRKs, NPR proteins involved in the SA synthesis pathway, and MAPKs, which were all potato late blight response proteins [23]. And the genes in plant interactions with pathogens, the MAPK signaling pathway and plant hormone signal transduction influence the resistance reaction of many crops moreover [24–26]. So we speculated that the up-regulated expression of these genes may be increasing resistance of the F scion to late blight. The Q/F *vs* Q comparison revealed relatively few DEGs. The identified up-regulated genes were mainly involved in the synthesis of the endoplasmic reticulum, endocytosis, and cell-to-cell movement. There were only 6 up-regulated genes were related to potato responses to late blight (3 *HSP90*, a *GSH-Px* and 2 *L-ascorbate peroxidase (APX)*). This may have been because Q is a disease-resistant variety in which these genes are already highly expressed. The expression of these genes may be related to the resistance of the Q scion to late blight. Therefore, we speculate that rootstock F has little effect on the late blight resistance of scion Q.

## Conclusions

The above-mentioned results indicate that the self-grafting of susceptible potato varieties and the grafting to disease-resistant varieties as the rootstock can increase the resistance of susceptible potato varieties to late blight, but the resistance is greater when resistant varieties are used as the rootstock. Using susceptible varieties as rootstocks had no significant effect on disease resistance of disease-resistant scion. And the late blight resistance is associated with the changes to the expression of resistance-related genes in the scion. Up-regulation of resistance gene expression can result in the late blight resistance of susceptible scions being increase. The DEGs identified in this study are potential candidate genes for future functional analyses. Moreover, the study findings may provide the basis for future investigations of the molecular mechanism underlying the enhanced disease resistance of scions resulting from potato xenografting.

## Methods

### Materials

The potato late blight-resistant variety Qingshu 9 and the susceptible variety Favorita were provided by the Biotechnology Institute of the Guizhou Academy of Agricultural Sciences, Guiyang city, Guizhou province, China. *Phytophthora infestans* W1 was provided by Guizhou University/Guizhou Provincial Biochemical Engineering Center, Guiyang city, Guizhou province, China.

### Methods

#### Grafting

For both varieties, virus-free potato blocks with only one bud eye were sown in an 11-cm diameter pot filled with sterile nutrient soil and then placed in a glass greenhouse at the Institute of Agricultural Bioengineering of Guizhou University on June 26, 2019. Plants were grafted on July 26, 2019 using the “splice method. Specifically, virus-free potato segments were placed in sterile nutrient soil. Uniformly growing 4-week-old plants with non-hollow branches were selected for grafting. Healthy young shoots (4–5 cm) with 4–5 leaves were used as scions, whereas healthy young shoots were cut 2–3 cm above the soil level to produce the rootstocks. During grafting, a 0.7-cm deep vertical incision was made in the middle of the rootstock. The scion was cut into wedges, inserted into the incision, and immediately covered with plastic wrap. Finally, the graft union was secured with a grafting clip, after which the seedlings were covered with plastic cups (Fig. 2b). The grafting procedure was completed in a glass greenhouse.

#### Evaluation of late blight resistance after grafting

Stably growing grafted plants were examined to assess their resistance to late blight, with the ungrafted F and Q plants sown on June 26, 2019 used as the controls. Late blight resistance was evaluated using the in vitro leaf inoculum method. Before inoculating the leaves, *P. infestans* cultured for 15 days was added to test tubes containing sterile water. The solution was then passed through 1–2 layers of filter paper, after which the filtrate was examined with a microscope to confirm the production of sporangia. The sporangia were placed in a refrigerator at 4 °C for 1 h to promote the release of zoospores until the concentration reached 2 × 10^4^ spores/μL. The healthy third leaf (from the top of the plants) was collected for inoculations. Leaf samples were collected from F and Q ungrafted controls and the F/F, Q/Q, Q/F, and F/Q grafted samples. 3 biological replicates were set for each grafting combination, with 2 leaves per replicate. The leaves were placed in plastic Petri dishes with the abaxial side facing up, after which they were covered with wet filter paper and sprayed with 2 mL distilled water. Using a pipette, the leaves were inoculated with a 20-μL *P. infestans* suspension. The inoculation site was located next to the main vein. The Petri dishes were sealed with Parafilm and then incubated at 22 °C with a 16-h light/8-h dark cycle. The leaves were checked for disease symptoms daily, with a particular focus on moisture retention. Symptoms were detectable at 5 days after the inoculation, and the size of the diseased area was measured on day 7. The longest and widest diseased spots were recorded [length (L) and width (W) being perpendicular], after which the lesion area was calculated using the following formula: A = 1/4 × π × L × W. According to the classification index proposed by Yao et al. (2001), late blight disease severity was assessed using the following levels [27]: level 1: no symptoms or the lesion area was less than 3%; level 2: the lesion area was between 3 and 10%, with no chlorosis and water immersion around the dead tissue; level 3: the lesion area was between 10 and 30%, and the surrounding area was soaked and contained white mycelia; level 4: the lesion area was between 30 and 60%, with obvious white mycelia; and level 5: the lesion area was greater than 60%, with obvious rotted tissue.

#### Transcriptome sequencing after grafting

On August 26, 2019, the third leaf from the top of uniformly growing and healthy plants (F/Q, Q/F, and ungrafted F and Q) was collected for the subsequent transcriptome sequencing analysis with the Illumina HiSeq 4000 high-throughput platform (Fig. 2a). The sequencing was completed with three biological replicates. The RNA extraction and transcriptome sequencing were performed by Beijing Nuohezhiyuan Technology Co., Ltd.

1. Identification of DEGs
  In order to compare the gene expression level of potato RNA library before and after grafting, featureCounts were used to calculate the reading of each gene and make a quantitative analysis of the gene expression level. After the quantitative analysis, statistical analysis of the expression data was performed to screen the genes whose expression levels were significantly different in the samples under different conditions. First, the original readcount was normalized, that is, the sequencing depth is corrected, then the statistical model was calculated for the hypothesis test probability (p-value), and finally the multiple hypothesis test was corrected to obtain the FDR value, that was, the false discovery rate, which was mainly padj. The selection criteria for DEGs were |log2(FoldChange)|> 1 & padj < 0.05. GO function enrichment and KEGG pathway enrichment were performed on DEGS, and the GO function and KEGG pathway meeting the P value of 0.05 standard were defined as significant enrichment of DEGs.
2. GO and KEGG analysis
  Clusterprofiler software was used to perform GO function enrichment analysis and KEGG pathway enrichment analysis. Based on potato genome database, gene function annotation was performed using cluster transcriptome sequences and public databases, and the differential genes were annotated into gene sets in GO or KEGG databases.

## Data Availability

The datasets used and/or analysed during the current study are available from the author on reasonable request.

## Abbreviations

KEGG: Kyoto Encyclopedia of Genes and Genomes
MAPK: Plant mitogen-activated protein kinase
CDPKs: Calcium-dependent protein kinases
CERKs: Chitin elicitor receptor kinases
LRR-LRKs: LRR receptor serine/threonine protein kinases
CNGC: Nucleotide-gated ion channel
SA: Salicylic acid
JA: Jasmonic acid
ET: Ethylene
Aux1: Auxin inlux carrier
SAUR: Small auxin-up RNA
HtpG: Heat shock protein
SRK2: Serine/threonine protein kinase
PP2C: Protein phosphatase
ABA: Abscisic acid
GO: Gene Ontology
BP: Biological process
CC: Cellular component
MF: Molecular function
DEGs: Differentially expressed genes
